# Identifying Influencers in Social Networks

**DOI:** 10.3390/e22040450

**Published:** 2020-04-15

**Authors:** Xinyu Huang, Dongming Chen, Dongqi Wang, Tao Ren

**Affiliations:** Software College, Northeastern University, Shenyang 110169, China; neuhxy@163.com (X.H.); wangdq@swc.neu.edu.cn (D.W.); rent@swc.neu.edu.cn (T.R.)

**Keywords:** complex network, social network analysis, multilayer network, node influence

## Abstract

Social network analysis is a multidisciplinary research covering informatics, mathematics, sociology, management, psychology, etc. In the last decade, the development of online social media has provided individuals with a fascinating platform of sharing knowledge and interests. The emergence of various social networks has greatly enriched our daily life, and simultaneously, it brings a challenging task to identify influencers among multiple social networks. The key problem lies in the various interactions among individuals and huge data scale. Aiming at solving the problem, this paper employs a general multilayer network model to represent the multiple social networks, and then proposes the node influence indicator merely based on the local neighboring information. Extensive experiments on 21 real-world datasets are conducted to verify the performance of the proposed method, which shows superiority to the competitors. It is of remarkable significance in revealing the evolutions in social networks and we hope this work will shed light for more and more forthcoming researchers to further explore the uncharted part of this promising field.

## 1. Introduction

The research of network science is experiencing a blossom in the last decade, which provides profound implications in very different fields, from finance to social and biological networks [1]. Considering the enormous data scale, most studies merely focus on a small group of influential nodes rather than the whole network. Take social networks for instance, influential nodes are those that have the most spreading ability, or playing a predominant role in the network evolution. Notably, a popular star in online social media may remarkably accelerate the spreading of rumors, and a few super spreaders [2] could largely expand the epidemic prevalence of a disease (e.g., COVID-19) [3]. The research of influencer identification is beneficial to understanding and controlling the spreading dynamics in social networks with diverse applications such as epidemiology, collective dynamics and viral marketing [4,5].

Nowadays, individuals interact with each other in more complicated patterns than ever. It is a challenging task to identify influencers in social networks for the various kinds of interactions. As we have known, the graph model is widely utilized to represent social networks, however, it is incapable of dealing with the multiple social links. For example, people use Facebook or WeChat to keep communication with family members or friends, use Twitter to post news, use LinkedIn to search for jobs, and use TikTok to create and share short videos [6]. It is easy to represent each social scenario via a graph model separately, in spite of they are belonging to the same group of individuals. The neglect of the multiple relationships between social actors may lead to an incorrect result of the most versatile users [7]. With the proposal of multilayer networks [8,9], we are able to encode the various interactions, which is of great importance and necessity of identifying influencers in multiple social networks.

In this paper, we design a novel node centrality measure for monolayer network, and then apply it to multilayer networks to identify influencers in multiple social networks. This method is solely based on the local knowledge of a network’s topology in order to be fast and scalable due to the huge size of networks, and thus suitable for both real-time applications and offline mining.

The rest of this paper is organized as follows. Section 2 introduces the related works on influencers identification in monolayer network and multilayer networks. Section 3 presents the mathematical model and the method for detecting influencers. Section 4 exhibits the experiments and analysis, including comparison experiments on twenty-one real-world datasets, which verifies the feasibility and veracity of the proposed method. Section 5 summarizes the whole paper and provides concluding remarks.

## 2. Related Works

The initial research on influencers identification may date back to the study of node centrality, which means to measure how “central” a focal node is [10]. A plethora of methods for influencers identification are proposed in the past 40 years, which can be mainly classified into centrality measures, link topological ranking measures, entropy measures, and node embedding measures [11,12]. Some of these measures take only the local information into account, while others even employ machine learning methods. Nowadays, it has been one of the most popular research topics and yielded a variety of applications [7] such as identifying essential proteins and potential drug targets for the survival of the cell [13], controlling the outbreak of epidemics [14], preventing catastrophic outages in power grids [15], driving the network toward a desired state [16], improving transport capacity [17], promoting cooperation in evolutionary games [18], etc. This paper investigates the problem of identifying influencers in social networks, by introducing a family of centrality-like measures and gives a brief comparison in Table 1.

Degree Centrality (DC) [19] is the simplest centrality measure, which merely counts how many social connections (i.e., the number of neighbors) a focal node has, defined as

(1) $$DC\left(i\right)=\sum_{j}^{N}a_{ij},$$

where *N* is the total number of nodes, $a_{ij}$ is the weight of edge (i,j) if *i* is connected to *j*, and 0 otherwise. The degree centrality is simple and merely considers the local structure around a focal node [20]. However, this method is probably mistaken for the negligence of global information, i.e., a node might be in a central position to reach others quickly although it is not holding a large number of neighbors [21]. Thus, Betweenness Centrality (BC) [22] is proposed to assess the degree to which a node lies on the shortest path between two other nodes, defined as

(2) $$BC\left(i\right)=\sum_{s\neq i,s\neq t,i\neq t}\frac{g_{st}\left(i\right)}{g_{st}},$$

where $g_{st}$ is the total number of shortest paths, $g_{st}\left(i\right)$ is the shortest path between *s* and *t* that pass through node *i*. The betweenness centrality considers global information and can be applied to networks with disconnected components. However, there is a great proportion of nodes that do not lie on the shortest path between any two other nodes, thereby the computational result receives the same score of 0. Besides, high computational complexity is also a limitation of applying for large-scale networks. Analogously, Closeness Centrality (CC) [23] is proposed to represent the inverse sum of shortest distances to all other nodes from a focal node, defined as

(3) $$CC\left(i\right)=\frac{N-1}{\sum_{j\neq i}d_{ij}},$$

where *N* is the total number of nodes, $d_{ij}$ is the shortest path length from node *i* to node *j*. The closeness centrality is capable of measuring the core position of a focal node via the utilization of global shortest path length, while it suffers from the lack of applicability to networks with disconnected components, e.g., if two nodes that belong to different components do not have a finite distance between them, it will be unavailable. Besides, it is also criticized by high computational complexity.

Eigenvector Centrality [24] (EC) is a positive multiple of the sum of adjacent centralities. Relative scores are assigned to all nodes in a network based on an assumption that connections to high-scoring nodes contribute more to the score of the node than connections to low-scoring nodes, defined as

(4) $$EC\left(i\right)=k_{1}^{-1}\sum_{j}A_{ij}x_{j},$$

where $k_{i}$ depicts the eigenvalue of adjacency matrix *A*, $x=k_{1}Ax$ depicts the eigenvector stable state of interactions with eigvenvalue $k_{1}^{-1}$. This measure considers the number of neighbors and the centrality of neighbors simultaneously, however, it is incapable of dealing with non-cyclical graphs. In 1998, Brin and Page developed the PageRank algorithm [25], which is the fundamental search engine mechanism of Google. PageRank (PR) is a positive multiple of the sum of adjacent centralities, defined as

(5) $$PR_{k}\left(i\right)=\sum_{j=1}^{N}a_{ji}\frac{PR_{k-1}\left(j\right)}{k_{j}^{out}},i=1,2,\ldots ,N.$$

where *N* depicts the total number of nodes, $\sum_{i=1}^{N}PR_{0}\left(i\right)=0$, $k_{j}^{out}$ is the number of edges from node *j* point to *i*. Likewise, this method is efficient but also criticized by non-convergence in cyclical structures. As we have known, the clustering coefficient [26,27] is a measure of the degree to which nodes in a graph tend to cluster together, defined as

(6) $$C_{i}=\frac{\sum_{j\neq i,k\neq j,k\neq i}a_{ij}a_{ik}a_{jk}}{\sum_{j\neq i,k\neq j,k\neq i}a_{ij}a_{ik}}.$$

It is widely considered that a node with a higher clustering coefficient may benefit forming communities and enhancing local information spreading. However, Chen et al. expressed contrary views that the local clustering has negative impacts on information spreading. They proposed a ClusterRank algorithm for ranking nodes in large-scale directed networks and verified its superiority to PageRank and LeaderRank [28]. Therefore, the effect of clustering coefficient on information spreading is uncertain, which may benefit local information spreading but prohibit global (especially directional network) information spreading. In 2016, Ma et al. proposed a gravity centrality [29] (GR) by considering the interactions comes from the neighbors within three steps, defined as

(7) $$G\left(i\right)=\sum_{j\in \psi_{i}}\frac{k_{s}\left(i\right)k_{s}\left(j\right)}{d_{ij}^{2}},$$

(8) $$G_{+}\left(i\right)=\sum_{j\in \Gamma_{i}}G\left(j\right),$$

where $k_{s}\left(i\right)$ and $k_{s}\left(j\right)$ are the *k*-shell index of *i* and *j*, respectively. $\psi_{i}$ is the neighborhood set whose distance to node *i* is less than or equal to 3, $d_{ij}$ is the shortest path length between *i* and *j*. These methods consider semi-local knowledge of a focal node, i.e., the neighboring nodes within three steps, which are successful in many real-world datasets, such as Jazz [30], NS [31] and USAir network [32], etc. However, they are also with high computational complexity by globally calculating *k*-shell. In 2019, Li et al. improved the gravity centrality and proposed a Local-Gravity centrality (LGR) [33] by replacing *k*-shell computing and merely considering the neighbors within *R* steps, defined as

(9) $$LG_{R}\left(i\right)=\sum_{d_{i}j\leq R,j\neq i}\frac{k_{i}k_{j}}{d_{ij}^{2}},$$

where $k_{i}$ and $k_{j}$ are the degrees of *i* and *j*, respectively, $d_{ij}$ is the shortest path length between *i* and *j*. This method had been extremely successful in a variety of real-world datasets, however, the parameter *R* requires the calculating of network diameter, which is also a time-consuming process.

The above-mentioned centrality measures have been utilized to rank nodes’ spreading abilities in monolayer networks. The ranking of nodes in multilayer networks is a more challenging task and is still an open issue. The information propagation process over multiple social networks is more complicated, and conventional models are incapable without any modifications. Zhuang and Yaǧan [36] proposed a clustered multilayer network model, where all constituent layers are random networks with high clustering to simulate the information propagation process in multiple social networks. Likewise, Basaras et al. [37] proposed an improved susceptible–infected–recovered (SIR) model with information propagation probability parameters (i.e., $\lambda_{ii}$ for intralayer connections and $\lambda_{ij}$ for interlayer connections). Most of the recent endeavors concentrated on the multiplex networks, (e.g., clustering coefficient in multiplex networks [38]), where all layers share the identical set of nodes but may have multiple types of interactions. Rahmede et al. proposed a MultiRank algorithm [39] for the weighted ranking of nodes and layers in large multiplex networks. The basic idea is to assign more centrality to nodes that are linked to central nodes in highly influential layers. The layers are more influential if highly central nodes are active in them. Wang et al. proposed a tensor decomposition method (i.e., EDCPTD centrality) [7], which utilize the fourth-order tensor to represent multilayer networks and identify essential nodes based on CANDECOMP/PARAFAC (CP) tensor decomposition. They also exhibited the superiority to traditional solutions by comparing the performance of the proposed method with the aggregated monolayer networks. In a word, it is of great significance in identifying influencers in multiplex networks. Our purpose in this work is to devise a measure that can accurately detect influential nodes in a general multilayer network.

## 3. Modeling and Methods

### 3.1. Network Modeling

The problem of finding influential nodes is described as extracting a small set of nodes that can bring the greatest influence on the network dynamics. With a given network model G=(V,E), where $V=\{v_{1},v_{2},\ldots ,v_{n}\}$ is the node set and $E=\{\left(v_{i},v_{j}\right)\}$, ($v_{i}$, $v_{j}\in V$) is the edge set. The identification of influential nodes is to pick a minimum of nodes as the initial seeds, which can achieve the maximum influenced scope, described as

(10) $$A=\arg\min_{A\in V}\max\left\{\sigma \left(A\right)\right\},$$

where *A* is the initially infected nodes, σ(A) denotes the final influenced node set. This problem is simplified as top-*k* influencers identification by additional setting |A|=k, which has recently attracted great research interests [40,41,42]. A variety of real-world social networks are, in fact, interconnected by different types of interactions between nodes, forming what is known as multilayer networks. In this paper, we employ a multilayer network model [9], which can represent nodes sharing links in different layers. The multilayer network model is defined as

(11) M=(G,C),

where $G=\{G_{\alpha };\alpha \in \left\{1,\ldots ,L\right\}\}$ is a family of (directed or undirected, weighted or unweighted) graphs $G_{\alpha }=\left(V_{\alpha },E_{\alpha }\right)$, which represents layers of M and C depicts the interactions between nodes of any two different layer, given by

(12) $$C=\{E_{\alpha \beta }\subseteq V_{\alpha }\times V_{\beta };\alpha ,\beta \in 1,\ldots ,L,\alpha \neq \beta \},$$

The corresponding supra-adjacency matrix can be represented as

(13) $$M=\left[\begin{array}{cccc} A_{1} & I_{12} & \cdots & I_{1L}\\ I_{21} & A_{2} & \cdots & I_{2L}\\ \vdots & \vdots & \ddots & \cdots \\ I_{L1} & I_{L2} & \cdots & A_{L} \end{array}\right]\in R^{N\times N},$$

where $A_{1},A_{2},\ldots ,A_{L}$ are the adjacency matrix of layer 1,2,…,L, respectively. *N* is the total number of the nodes, which can be calculated by $N=\sum_{1\leq l\leq L}\left|V^{l}\right|$. The non-diagonal block $I_{\alpha \beta }$ represents the inter-layer edges of layer α and layer β. Thus, the interlayer edges can be represented as

(14) $$I=\overset{L}{\underset{\alpha ,\beta =1,\alpha \neq \beta }{⋃}}I_{\alpha \beta }.$$

**Figure 1:**
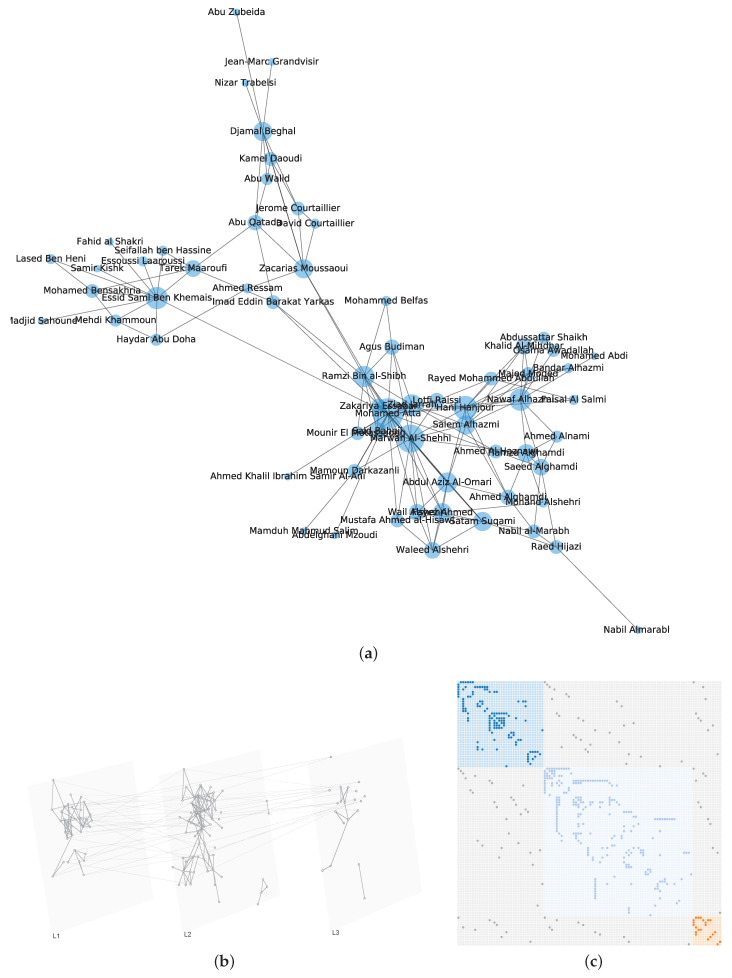
Network of 9/11 terrorists. (**a**) The monolayer network representation, the size of a node represents its degree; (**b**) The 9/11 terrorists’ interactions represented by a multilayer network model, where $L_{1}$ presents confirmed close contact, $L_{2}$ layer shows various recorded interactions, $L_{3}$ contains potential or planed or unconfirmed interactions; (**c**) The super-adjacency matrix representation.

Take the 9/11 terrorists network [43] for instance, the edges are classified into three categories (i.e., layers) according to the observed interactions which are plotted in Figure 1.

### 3.2. Methods

We employ the susceptible–infected–recovered (SIR) spreading model [44] as the influence analysis model. It has three possible states:

- Susceptible (S) state, where a node is vulnerable to infection.
- Infectious (I) state, where a node tries to infect its susceptible neighbors.
- Recovered (R) state, where a node has recovered (or isolated) and can no longer infect others.

**Figure 2:**
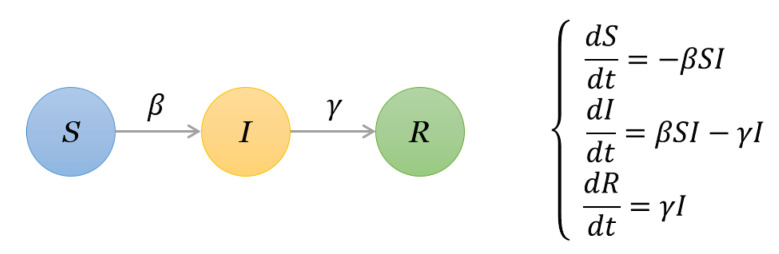
The illustration of parameters in the susceptible–infected–recovered (SIR) model and corresponding differential equations.

**Figure 3:**
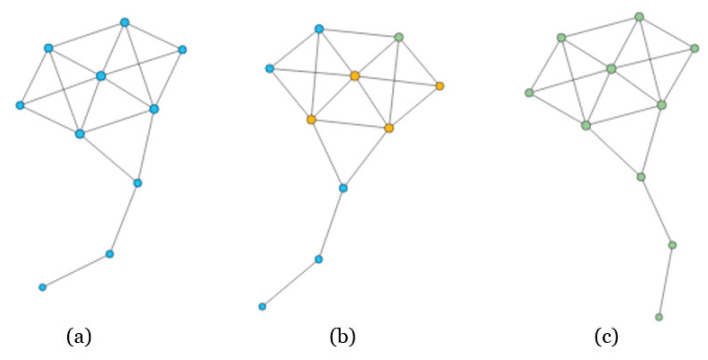
The process of SIR model on Krackhardt’s Kite network. In panel (**a**), all the nodes are in Susceptible state; while we select one node to be infected, and the neighbors will be infected soon, as shown in panel (**b**); Finally, the network will reach a stable state, i.e., the number of recovered nodes will reach a maximum, as shown in panel (**c**).

**Figure 4:**
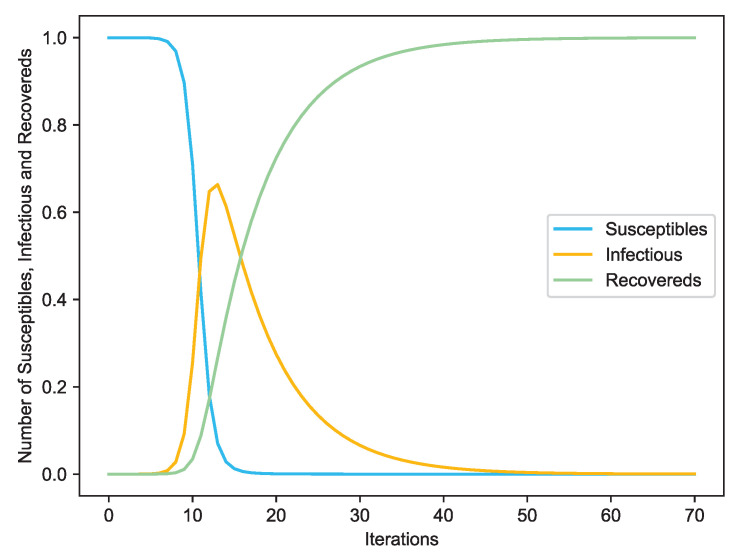
The varying susceptible, infectious and recovered nodes with the increasing of iterations.

In a network, if two nodes are connected then they are considered to have “contact”. If one node is “infected”, and the other is susceptible, then with a certain probability the latter may become infected through contact [45]. A node is considered to be recovered if it is isolated or immune to the disease. In detail, to check the spreading influence of one given node, we set this node as an infected node and the other nodes are susceptible nodes. At each time step, each infected node can infect its susceptible neighbors with infection probability β, and then it recovered from the diseases with probability γ, the differential equations are shown in Figure 2. For simplicity, we set γ=1. The process of the SIR model is plotted in Figure 3 and Figure 4 with the famous Krackhardt’s Kite network [46].

In this paper, we define the node influence (INF, for short) as the energy derived from the neighbors, given as

(15) $$INF_{R}\left(i\right)=\sum_{d_{ij}\leq r}\sum_{j\in \Gamma (i)}\frac{d_{ij}^{2}w_{ij}}{k_{j}},$$

where *R* is the truncation radius, Γ(i) is the set of neighbors of node *i*, $d_{ij}$ is the shortest path length between node *i* and node *j*, $k_{j}$ is the degree of node *j*, $w_{ij}$ is the weight of edge $e_{ij}$. For unweighted networks, $w_{ij}=1$. Analogously, we apply the proposed INF measure to multilayer networks (represented as $INF_{R}^{M}$) by the following modifications

(16) $$INF_{R}^{M}\left(i\right)=\sum_{d_{ij}\leq R}\sum_{\alpha <L}\sum_{j\in \Gamma^{\alpha }\left(i\right)}\frac{d_{ij}^{2}w_{ij}}{k_{j}^{\alpha }},$$

where *R* is the truncation radius, $\Gamma^{\alpha }\left(i\right)$ is the set of neighbors of node *i* at layer α, $k_{j}^{\alpha }$ is the degree of node *j* at layer α, $d_{ij}$ is the shortest path length between node *i* and node *j*. For simplicity, we choose R=1, thus $d_{ij}=1$ if node *i* and node *j* is connected through an intralayer edge or interlayer edge, and 0 otherwise.

**Figure 5:**
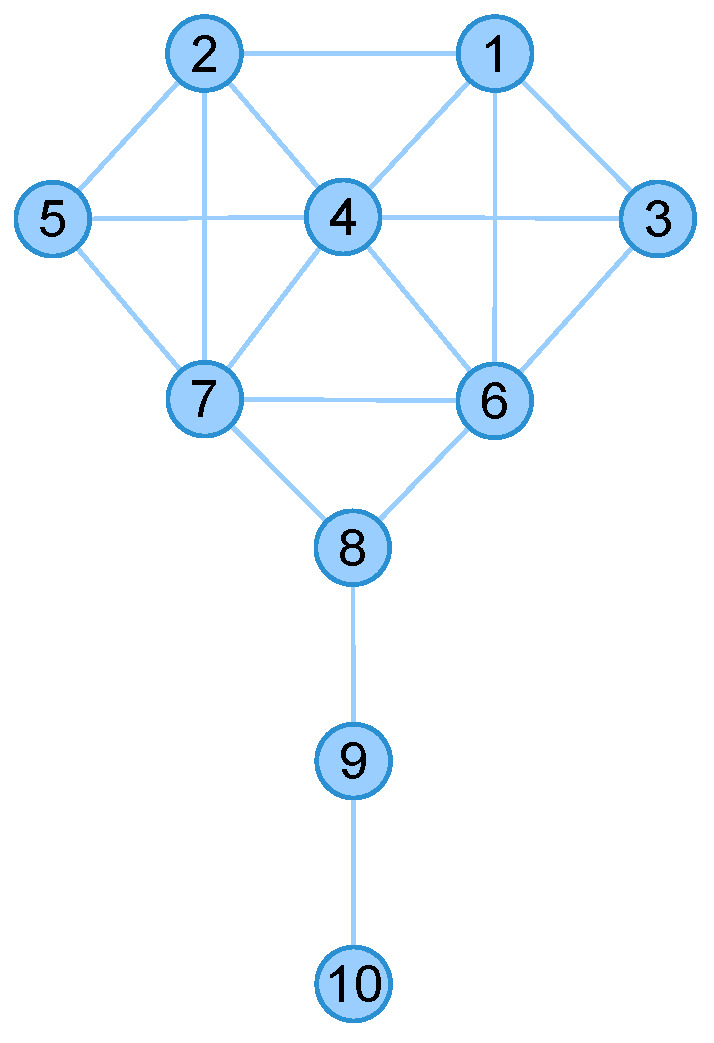
The network structure of Krackhardt’s Kite network.

To explain the effect, we take the above-mentioned Krackhardt’s Kite network (as plotted in Figure 5) and the 9/11 terrorists network (as plotted in Figure 1) for examples. The nodes centralities in Krackhardt’s Kite network are shown in Table 2.

As shown in Table 2, Node 4 is considered to be the most important node under the Degree, Katz and the proposed INF measure, while Node 8 has greater Betweenness, Node 6 and node 7 has greater Closeness or (Eigenvector). Thus, the node list (i.e., [4, 6, 7, 8]) is considered to be the influencers. Furthermore, to evaluate the nodes’ influence, we set each node as the initially infected and recorded the final recovered nodes, respectively. This process is repeated for 10,000 times and the results are shown in Table 3.

**Figure 6:**
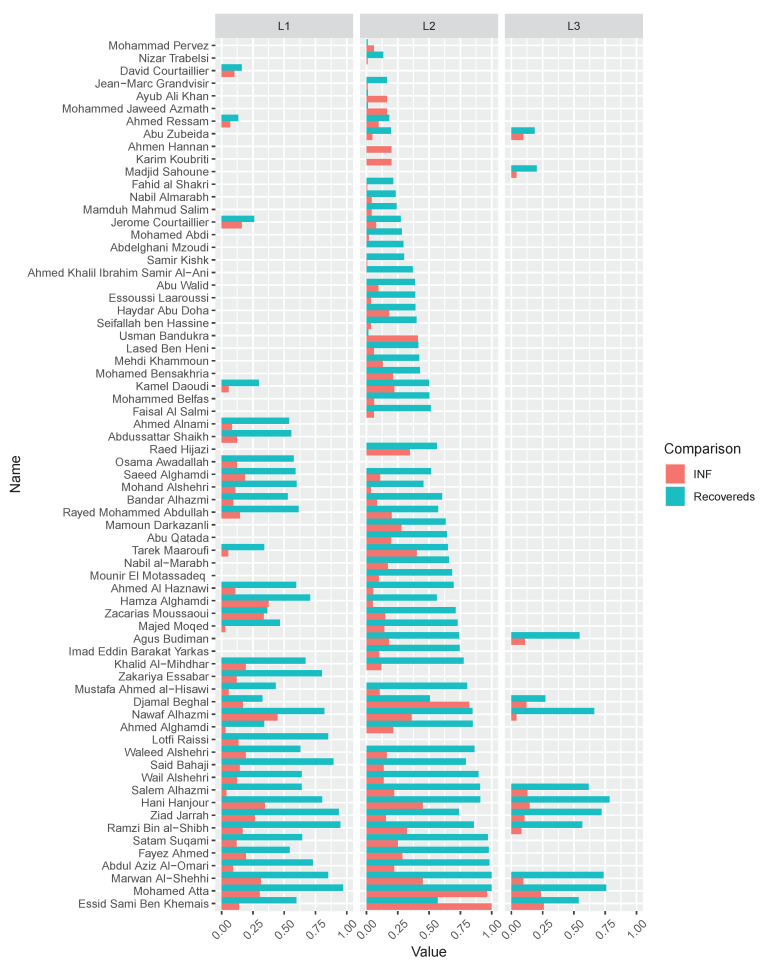
The comparison of recovered nodes in 9/11 terrorists network by initially setting every node infected separately. The recovered nodes and the corresponding INF values are normalized, which are marked in cyan and red, respectively. The comparison of the nodes in three layers are plotted in facet $L_{1}$,$L_{2}$ and $L_{3}$.

As shown in Table 3, Node 4 (i.e., Diane), which is considered to be more influential under Degree, Katz, and INF centrality, shows more recovered nodes (i.e., 5.3182) after 10,000 times SIR stimulations. This experiment is available at https://neusncp.com/api/sir. Analogously, we conduct experiments on the three-layer 9/11 terrorists network. Particularly, we set the infected probability between intralayer edges as β and the probability between interlayer edges as $\beta^{M}=w_{ij}\beta$. The experimental results are plotted in Figure 6.

By conducting SIR simulations on the three-layer 9/11 terrorists network, we can obtain the influential nodes of each layer by calculating the number of finally recovered nodes. Afterward, we sort the nodes by the averaging recovered nodes, and compare the order with the results computed from the proposed INF indicator. It is shown in Figure 6 that the compared values (i.e., recovered nodes and INF) are in the same tendency, which verifies the feasibility of the proposed INF measure. Notably, several influential nodes, such as “Essid Sami Ben Khemais”, “Mohamed Atta”, and “Marwan Al-Shehhi” are also in the central position of the network, as shown in Figure 1a.

The experimental results on the two sample networks show the feasibility of the proposed measure on monolayer and multilayer networks, respectively. Experiments on more real-world networks will be given in Section 4.

### 3.3. Complexity Analysis

Suppose *m* and *n* are the numbers of edges and nodes, respectively, *L* is the number of layers, the average degree of nodes is *d*, *R* is the truncation radius (commonly setting as R=1). The complexity of INF for monolayer network is $O(n+d^{R})$. As for multilayer networks, the computational complexity is $O(n+Ld^{R})$, where *L* is also a small positive integer. Thus, the time complexity is acceptable as O(n+Ld). Overall, the proposed measure considers more neighboring information than the degree centrality and has a lower computational complexity than betweenness centrality and closeness centrality (i.e., $O(nm+n^{2}\log n)$).

## 4. Experiments and Discussion

The experimental environment was with Intel(R) Core (TM) i5-7200U CPU @ 2.50 GHz (4 CPUs), 2.7 GHz, the memory was 8 GB DDR3. The operating system was Windows 10 64 bit, the programming language was Python 3.7.1, and the relevant libs were NetworkX 2.2 and Multinetx. The goal of the experiments was to compare the performance of the proposed INF measure with competitive indicators.

### 4.1. Experimental Datasets

In this paper, 21 real-world datasets were employed to verify the performance of the proposed method, which were classified into two groups. The first group covered 12 monolayer networks, which comprised four social networks (i.e., Club, Dolphins, 911 and Lesmis), three biological networks (i.e., Escherichia, C.elegans and DMLC), collaboration networks (i.e., Jazz and NS), a communication network (i.e., Eron), a power network (i.e., Power) and a transport network (i.e., USAir), as shown in Table 4.

The second group covered nine multilayer networks, which comprised six social networks (i.e., Padgett, Krackhardt, Vickers, Kapferer, Lazega and CS-Aarhus), two transport networks (i.e., LondonTransport and EUAirTransportation) and a biological network (i.e., humanHIV), as shown in Table 5. Data availability: http://www.neusncp.com/user/file?id=12&code=data.

### 4.2. Performance Comparison

**Figure 7:**
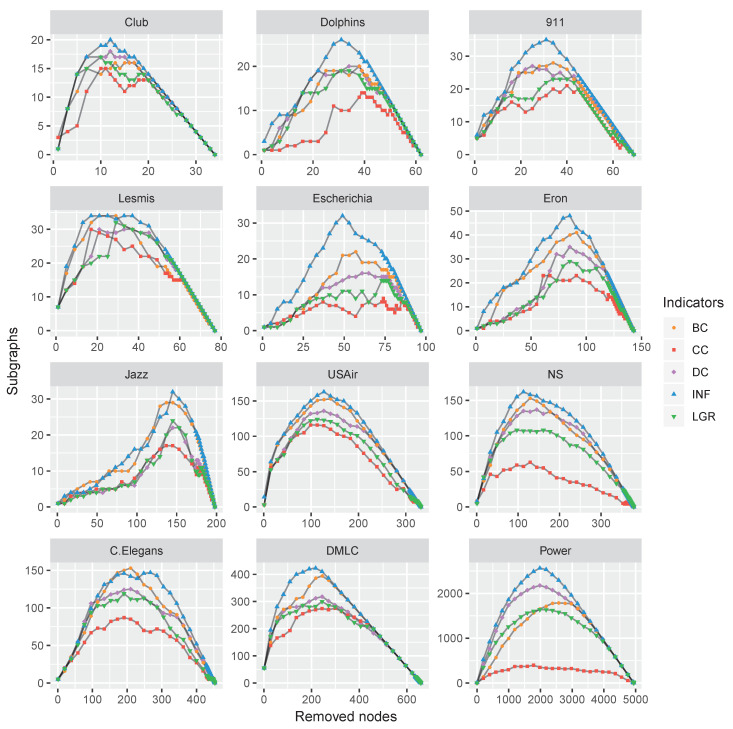
The varying subgraphs of 12 monolayer networks with the removal of the most influential nodes repeatedly with each centrality indicator.

To verify the performance of the proposed node influence in networks, this paper carries out a comparison experiment on the above-mentioned datasets: The nodes were removed by a certain indicator in descending order, and the number of subgraphs was recorded. This process repeated until there were not any nodes left. The varying tendency of the subgraphs’ number exhibited the influence of a focal centrality. The experimental results are shown in Figure 7.

As shown in Figure 7, with the nodes removing, the number of subgraphs was increasing and reached a maximum when the network was totally broken up, i.e., there were no edges at this moment. Afterward, the number of subgraphs (i.e., the number of nodes) was decreasing and finally reached zero when all the nodes were removed. The maximum numbers of subgraphs were obtained by the proposed INF measure on all the datasets except C.Elegans. However, the result of C.Elegans obtained by INF was very close to the best situation of BC, which suggests the feasibility of the proposed INF measure.

We applied the SIR model to compare the rankings of influences calculated by each indicator among the above-mentioned networks. Initially, one node was set as “infected” state to infect its neighbors with probability β. Afterward, the infected nodes were recovered and never be infected again with probability γ. This spreading process repeated until there were no more infected nodes in the network. The influence of any node *i* can be estimated by

(17) $$P\left(i\right)=\frac{N_{R}}{N},$$

where $N_{R}$ is the number of recovered nodes after the spreading process, and *N* is the total number of nodes in the network. For simplicity, we set γ=1 and the epidemic threshold was

(18) $$\beta_{c}\approx \frac{\langle k\rangle }{\langle k^{2}\rangle -\langle k\rangle }$$

After having obtained the standard nodes’ influence sequence via SIR model simulations, we employed the Kendall’s Tau coefficient [65] to compare the performance of each indicator. The Kendall’s Tau coefficient is an index measuring the correlation strength between two sequences. Suppose given the standard sequence $X=(x_{1},x_{2},\ldots ,x_{N})$, and we obtained the computational sequence $Y=(y_{1},y_{2},\ldots ,y_{N})$ by a certain indicator. Any pair of two-tuples $\left(x_{i},y_{i}\right)$ and $\left(x_{j},y_{j}\right)$ (x≠j) are concordant if both $x_{i}>x_{j}$ and $y_{i}>y_{j}$ or $x_{i}<x_{j}$ and $y_{i}<y_{j}$. Meanwhile, they are considered as discordant, if $x_{i}>x_{j}$ and $y_{i}<y_{j}$ or $x_{i}<x_{j}$ and $y_{i}>y_{j}$. If $x_{i}=x_{j}$ or $y_{i}=y_{j}$, pairs are neither concordant nor discordant. Therefore, Kendall’s Tau coefficient is defined as

(19) $$\tau =\frac{N_{c}-N_{d}}{0.5n(n-1)},$$

where $N_{c}$ and $N_{d}$ indicate the number of concordant and discordant pairs, respectively. The range of τ is [−1,1]. Table 6 shows the computational Tau results with the comparison of standard sequence from SIR model simulations.

As shown in Table 6, the proposed measure outperformed the competitors in most cases, even in the Escherichia network, the computed Tau result of INF (0.0692) was close to that of CC (0.0971). Thus, it was also competitive in this network.

**Figure 8:**
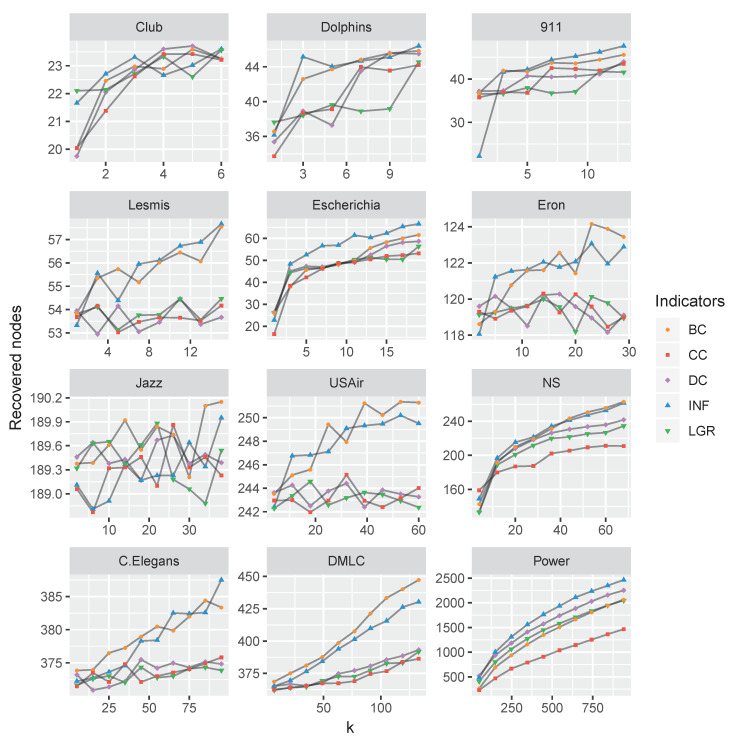
The algorithms’ performance comparison for varying *k* (ranging from 0 to 20 percent of total nodes), measured by the recovered nodes. The betweenness centrality and the proposed INF measure are very competitive than the others.

If setting the limitation of identifying *k* influencers, we conducted experiments on the real-world datasets with top-*k* nodes by computational centrality nodes and compared the recovered nodes (i.e., the final number of nodes with recovered states). To compare the varying parameter *k* with the obtained τ, we conducted experiments on the above-mentioned datasets and set the ratio of $\beta /\beta_{c}$, as shown in Figure 8.

As shown in Figure 8, the proposed node influence method is quite competitive in most of the datasets, although second to the performance of betweenness indicator in DMLC and Jazz datasets.

**Figure 9:**
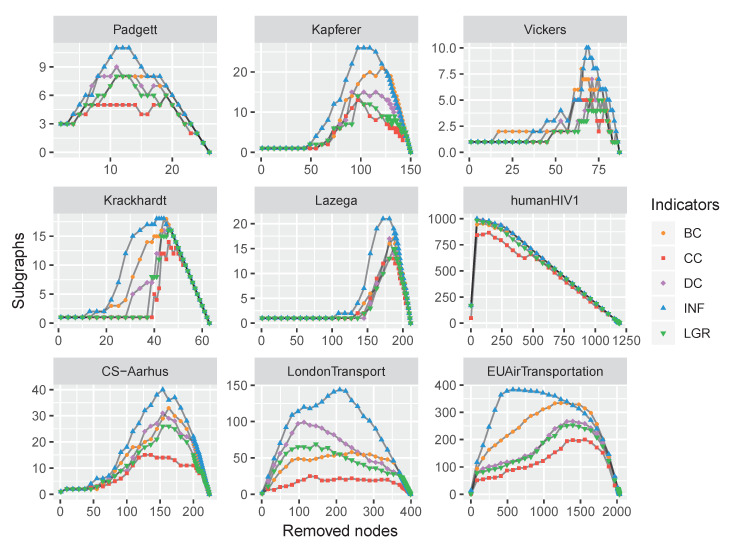
The computational time comparison of different indicators. The accumulation of running time on the 12 real-world datasets has exhibited that the proposed INF measure is much more efficient than the competitors.

Analogously, we conducted experiments on the nine multilayer networks by removing nodes with maximum centralities; the results are plotted in Figure 9.

**Figure 10:**
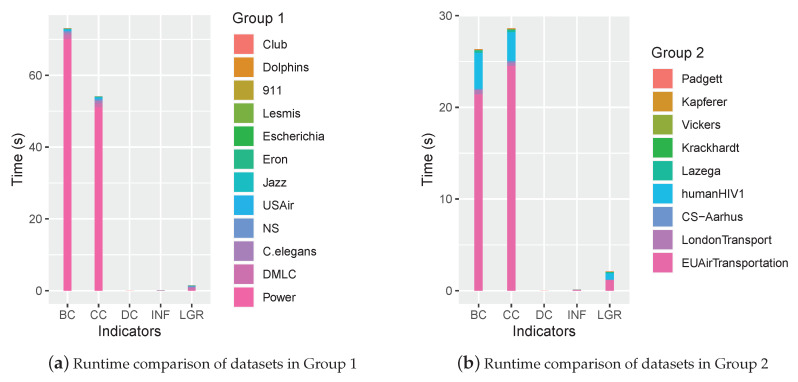
The runtime comparison of each indicator.

To compare the time complexity of the proposed INF measure with classic methods, the runtime of 21 networks are recorded and shown in Figure 10.

As shown in Figure 10, the runtime accumulated from either group indicated that the proposed INF measure was efficient, which was close to that of DC and superior to BC, CC and LGR.

### 4.3. Discussion

Influencers identification is a fundamental issue with wide applications in different fields of reality, such as epidemic control, information diffusion, viral marketing, etc. Currently, degree centrality [19] is the simplest method, which considers nodes with larger degrees are more influential. However, for the lack of global information, a node lying in a “bridge” position might be neglected for holding a small degree. The betweenness [22] and closeness [23] centrality consider global information, but they are holding a high complexity, which are not suitable for applications in large-scale networks. Local gravity is a balanced method, however, the determination of parameter *R* requires computing network diameter, which is also time-consuming. Thus, a novel node influence measure is proposed in this paper, which merely considers the local neighboring information of a focal node with the complexity of O(n+Ld). Experimental results on 21 real-world datasets indicate the feasibility of the proposed measure.

Firstly, the experiments of counting subgraphs with removing influential nodes show that the capability of the proposed INF measure. By removing the nodes according to the INF indicator, the networks are more easily broken up, as shown in Figure 7 and Figure 9. Secondly, we apply the SIR model to evaluate the node influence, which suggested the proposed INF measure is competitive to other indicators in most cases. Although inferior to BC on Jazz and DMLC networks, it is also competitive. By analyzing the structures of these two networks, we find that the nodes of Jazz network are densely connected (i.e., the average degree of 27.6970) and most of the nodes are holding the same number of neighbors (approximately 28 neighbors), which brings difficulties to identify which node is more influential. On the contrary, there is only one node (i.e., Node 2) holding a large number of neighbors (i.e., 439 neighbors) and the others only holding few neighbors (approximately four neighbors) in DMLC network, which is also difficult to identify influencers. Overall, the proposed method outperforms the other indicators in most cases. Finally, we compare the running time of each indicator on the 21 real-world datasets. Experimental results show the efficiency of the proposed measure.

## 5. Conclusions

Aiming at solving the problem of identifying influencers in social networks, this paper proposes a novel node influence indicator. This method merely considers the local neighboring information in order to be fast and suitable for applications in large-scale networks. Extensive experiments on 21 real-world datasets are conducted, and the experimental results show that the proposed method outperforms competitors. Afterwards, the time complexity is compared, and we verify the efficiency of the proposed indicator. Overall, the proposed node influence indicator is capable of identifying influencers in social networks. The contribution of this work is likely to benefit many real-world social applications, such as promoting network evolutions, preventing the spreading of rumors, etc.

As part of future works, the influencers in dynamic networks can be further studied by applying the proposed INF measure into a multilayer network model with numerous ordinal layers. The node’s influence can be calculated by accumulating the local neighbors across all the layers. Besides, the effect of layers needs to be taken into consideration. In a word, we hope the findings in this work will help to improve the researches in this promising field.

## Figures and Tables

**Table 1:** Classical node centrality metrics comparison.

Metric	Topology	Complexity	Advantages	Disadvantages
DC [19]	Local	O(n)	simple	incapable of dealing with “bridge” nodes
BC [22]	Global	$O(nm+n^{2}\log n)$	finding “bridge-like” nodes	cannot differentiate most marginal nodes
CC [23]	Global	$O(nm+n^{2}\log n)$	finding “nearest” nodes	incapable in disconnected graphs
EC [24]	Global	O(n+m)	consider both of the quality and quantity of neighbors	may be non-convergent
PR [25]	Global	O(n+m)	efficient, widely applied in search engine	may be non-convergent
H-index [34]	Semi-local	O(n+m)	famous for academic evaluation	lack of global information
*k*-shell [14]	Global	O(n+m)	suitable for large-scale networks	indistinguishable
LR [35]	Global	O(n+m)	no parameters; robustness	may be non-convergent
GR, GR+ [29]	Global	$O(n^{3})$	accuracy	high complexity in *k*-shell
LGR [33]	Semi-local	$O(n^{2})$	simple and capable in most cases	additional parameters *R* determination

**Table 2:** Classic node centralities comparison of kite network. The maximum centralities are marked in bold.

ID	Name	Degree	Betweenness	Closeness	Katz	Eigenvector	INF
1	Andre	0.4444	0.0231	0.5294	0.3307	0.3522	0.7211
2	Beverley	0.4444	0.0231	0.5294	0.3307	0.3522	0.7211
3	Carol	0.3333	0.0000	0.5000	0.3006	0.2858	0.6495
4	Diane	**0.6667**	0.1019	0.6000	**0.3907**	0.4810	**0.8273**
5	Ed	0.3333	0.0000	0.5000	0.3006	0.2858	0.6495
6	Fernando	0.5556	0.2315	**0.6429**	0.3595	**0.3977**	0.7830
7	Garth	0.5556	0.2315	**0.6429**	0.3595	**0.3977**	0.7830
8	Heather	0.3333	**0.3889**	0.6000	0.2887	0.1959	0.7109
9	Ike	0.2222	0.2222	0.4286	0.2431	0.0481	0.7914
10	Jane	0.1111	0.0000	0.3103	0.2168	0.0112	0.6225

**Table 3:** Averaging recovered nodes and iterations times of each node as initially infected spreaders under 10,000 times SIR stimulations with parameters setting β=0.35, γ = 1.

ID	Name	Recovered Nodes	Iterations
1	Andre	4.8015	3.3399
2	Beverley	4.7902	3.3392
3	Carol	4.3485	3.1529
4	Diane	**5.3182**	3.3684
5	Ed	4.3336	3.1455
6	Fernando	5.0856	3.3251
7	Garth	5.0338	3.2994
8	Heather	4.0765	2.9625
9	Ike	2.6060	2.1806
10	Jane	1.8086	1.7019

**Table 4:** Statistics of 12 real-world monolayer networks.

Dataset Name	|V|	|*E*|	<*k*>	<*d*>	|*C*|	*r*	|*H*|	$\beta_{c}$
Club [47]	34	78	4.5882	2.4082	0.5706	−0.4756	1.6933	0.1477
Dolphins [48]	62	159	5.1290	3.3570	0.2590	−0.0436	1.3268	0.1723
911 [49]	69	159	4.6087	2.4672	0.4698	−0.0380	1.7304	0.1434
Lesmis [50]	77	254	6.5974	2.6411	0.5731	−0.1652	1.8273	0.0905
Escherichia [51]	97	212	4.3711	5.5369	0.3675	0.4116	1.2367	0.2270
Eron [52]	143	623	8.7133	2.9670	0.4339	−0.0195	1.4829	0.0839
Jazz [30]	198	2742	27.6970	2.2350	0.6175	0.0202	1.3951	0.0266
USAir [32]	332	2126	12.8072	2.7381	0.6252	−0.2079	3.4639	0.0231
NS [31]	379	914	4.8232	6.0419	0.7412	−0.0817	1.6630	0.1424
C.elegans [53]	453	2032	9.0066	2.6638	0.6465	−0.2197	4.4782	0.0254
DMLC [54]	659	1570	4.7648	2.6370	0.3279	−0.1914	14.8897	0.0143
Power [26]	4941	6594	2.6691	18.9892	0.0801	0.0035	1.4504	0.3483

**Note:**|V| and |E| denotes the number of nodes and edges, respectively. <*k*> is the average degree; <*d*> is the average shortest path length; |C| is the average clustering index; <*r*> is the assortativity coefficient; |H| is the degree heterogeneity and $\beta_{c}$ represents the epidemic threshold of the SIR model. **Club** contains the friendships between the 34 members of a karate club at a US university. **Dolphins** dataset is a animals social network. **911** represents a monolayer terrorist network of September 11 attacks. **Lesmis** is the coappearance network of characters in the novel Les Miserables. **Escherichia** represetns transcriptional regulation networks in cells orchestrate gene expression, where nodes are operons, and each edge is directed from an operon that encodes a transcription factor to an operon that it directly regulates (an operon is one or more genes transcribed on the same mRNA). **Eron** is a email network collected from Eron company. **Jazz** lists the collaboration patterns of jazz musicians. **USAir** is an undirected weighted network as obtained by considering the 500 US airports with the largest amount of traffic from publicly available data. Nodes represent US airports and edges represent air travel connections among them. **NS** represents coauthorships between 379 scientists whose research centers on the properties of networks of one kind or another. **C.elegans** represents the edges of the metabolic network of C.elegans. **DMLC** represents the inferred Links by small/medium-scale rotein-protein interactions (collected from protein-protein interaction data bases). **Power** is a power grid of the western United States.

**Table 5:** Statistics of nine real-world multilayer networks.

Dataset Name	|L|	|V|	|*E*|	|$E_{A}$|	|$E_{C}$|	<*k*>	<*d*>	|*C*|
Padgett [55]	2	26	46	35	11	3.5385	2.6923	0.1441
Krackhardt [56]	3	63	307	244	63	9.746	2.1731	0.3943
Vickers [57]	3	87	605	518	87	13.908	2.1802	0.4823
Kapferer [58]	4	150	769	552	217	10.2533	2.5889	0.3002
Lazega [59,60]	3	211	2051	1842	209	19.4408	2.3958	0.3938
humanHIV1 [61]	5	1195	1504	1269	235	2.5172	4.1385	0.0221
CS-Aarhus [62]	5	224	948	620	328	8.4643	3.1847	0.3603
LondonTransport [63]	3	399	472	441	31	2.3659	14.2989	0.0243
EUAirTransportation [64]	37	2034	15199	3588	11611	14.9449	3.5087	0.5969

**Note:**|L| denotes the number of layers; |V| and |E| are the total number of nodes and edges, respectively; $|E_{A}|$ and $|E_{C}|$ denote the number of intralayer edges and interlayer edges, respectively. <*k*> is the average degree; <*d*> is the average shortest path length; |C| is the average clustering index; **Padgett** consists of 2 layers (marriage alliances and business relationships) describing florentine families in the Renaissance; **Krackhardt** consists of 3 kinds of relationships (Advice, Friendship and “Reports to”) between managers of a high-tech company; **Vickers** is collected by Vickers from 29 seventh grade students in a school in Victoria, Australia. Students are asked to nominate their classmates on a number of three kinds of relations; **Kapferer** exhibits interactions in a tailor shop in Zambia (then Northern Rhodesia) over a period of ten months, where layers represent two different types of interaction, recorded at two different times (seven months apart) over a period of one month; **Lazega** consists of three kinds of interactions (Co-work, Friendship and Advice) between partners and associates of a corporate law partnership; **humanHIV1** represents the multiplex genetic and protein interactions network of the human HIV type 1; **CS-Aarhus** consists of five kinds of online and offline relationships (Facebook, Leisure, Work, Co-authorship, Lunch) between the employees of Computer Science department at Aarhus; **LondonTransport** is collected from the official website of Transport for London (https://www.tfl.gov.uk/). Nodes are train stations in London and edges encode existing routes between stations; **EUAirTransportation** is composed by thirty-seven different layers each one corresponding to a different airline operating in Europe.

**Table 6:** The indicators’ accuracies measured by the Kendall’s Tau (τ).

Dataset Name	DC	INF	LGR	BC	CC
Club	0.2442	**0.2513**	0.1515	0.1016	−0.0766
Dolphins	0.0238	**0.0344**	−0.0196	−0.0323	−0.0354
911	0.0878	**0.1918**	0.0426	0.0409	−0.0895
Lesmis	0.0909	**0.1114**	0.1032	−0.1839	−0.0519
Escherichia	0.0726	0.0692	0.0009	−0.0808	**0.0971**
Eron	0.0454	**0.0927**	−0.0056	0.0031	0.0107
Jazz	0.069	**0.0838**	0.0325	0.0244	0.0312
USAir	−0.0358	**0.0743**	−0.0402	−0.0491	−0.0311
NS	−0.0378	**0.0302**	−0.0089	−0.0143	−0.0462
C.elegans	0.0154	**0.0697**	0.0242	0.0185	0.0214
Power	−0.005	**0.0272**	0.0024	−0.0015	0.0209

**Note:** Given a network, the parameters of SIR model are given with the transmission probability β=0.35 and recovering probability μ=1 for simplicity. To obtain the standard ranking of nodes’ influences, we conducted 1000 independent simulations, in each process every node is selected once as the infect seed once. The best perfromed indicator for each network is emphasized by bold.
